# Prediction of gene expression-based breast cancer proliferation scores from histopathology whole slide images using deep learning

**DOI:** 10.1186/s12885-024-13248-9

**Published:** 2024-12-11

**Authors:** Andreas Ekholm, Yinxi Wang, Johan Vallon-Christersson, Constance Boissin, Mattias Rantalainen

**Affiliations:** 1 Department of Medical Epidemiology and Biostatistics, Karolinska Institutet, Box 281, Stockholm, 171 77 Sweden; 2 Division of Oncology, Department of Clinical Sciences Lund, Lund University, Lund, Sweden; 3 MedTechLabs, BioClinicum, Karolinska University Hospital, Stockholm, Sweden

**Keywords:** Artificial intelligence, Computational pathology, Proliferation, Gene expression, Breast cancer

## Abstract

**Background:**

In breast cancer, several gene expression assays have been developed to provide a more personalised treatment. This study focuses on the prediction of two molecular proliferation signatures: an 11-gene proliferation score and the MKI67 proliferation marker gene. The aim was to assess whether these could be predicted from digital whole slide images (WSIs) using deep learning models.

**Methods:**

WSIs and RNA-sequencing data from 819 invasive breast cancer patients were included for training, and models were evaluated on an internal test set of 172 cases as well as on 997 cases from a fully independent external test set. Two deep Convolutional Neural Network (CNN) models were optimised using WSIs and gene expression readouts from RNA-sequencing data of either the proliferation signature or the proliferation marker, and assessed using Spearman correlation (r). Prognostic performance was assessed through Cox proportional hazard modelling, estimating hazard ratios (HR).

**Results:**

Optimised CNNs successfully predicted the proliferation score and proliferation marker on the unseen internal test set (*ρ* = 0.691(*p* < 0.001) with R^2^ = 0.438, and *ρ* = 0.564 (*p* < 0.001) with R^2^ = 0.251 respectively) and on the external test set (*ρ* = 0.502 (*p* < 0.001) with R^2^ = 0.319, and *ρ* = 0.403 (*p* < 0.001) with R^2^ = 0.222 respectively). Patients with a high proliferation score or marker were significantly associated with a higher risk of recurrence or death in the external test set (HR = 1.65 (95% CI: 1.05–2.61) and HR = 1.84 (95% CI: 1.17–2.89), respectively).

**Conclusions:**

The results from this study suggest that gene expression levels of proliferation scores can be predicted directly from breast cancer morphology in WSIs using CNNs and that the predictions provide prognostic information that could be used in research as well as in the clinical setting.

**Supplementary Information:**

The online version contains supplementary material available at 10.1186/s12885-024-13248-9.

## Background

Breast cancer, the most common cancer globally [1], is a very heterogeneous disease where prognosis is based not only on morphology and invasiveness, but also on molecular differences [2]. Clinical treatment decisions and prognostic predictions are performed by pathologists using either Haematoxylin and Eosin (H&E) stained slides of the resected tumour specimen to assign tumour grade according to the Nottingham Histologic Grade (NHG) system [3], or using immunohistochemically (IHC) stained slides to identify specific biomarkers. Several gene signature profiles have also been developed to classify patients into four intrinsic molecular subtypes (Luminal A, Luminal B, Basal-like and HER2-enriched) each with different treatment and prognostic significance [4, 5]. The benefit of these gene expression assays is that they more precisely characterise individual tumours, potentially leading to a more personalised treatment [6]. In addition, they may also reduce the high inconsistencies that can be seen between laboratories and pathologists in routine pathology [7, 8]. On the other hand, these assays have some limitations including that they involve a high number of genes, for example the PAM50 gene set is based on expression levels of 50 genes [4, 9], leading to significant costs and long lead times to obtain results.

Several attempts have been made to identify a smaller gene set while keeping the clinical relevance of the gene expression assays [2, 9, 10]. A combination of eleven proliferation-associated genes – a known feature of cancer cells [11] – from the original PAM50 assay has been suggested as an 11-gene proliferation score [4, 12]. Similarly, the gene MKI67, coding for the Ki-67 protein, is over-expressed during cell proliferation and both gene expression levels and protein expression in IHC have been shown to be linked with proliferation and more aggressive tumour behaviours [13–16].

Computational pathology, where deep learning, for example, Convolutional Neural Networks (CNNs), is used to model digitised H&E Whole Slide Images (WSIs) and has already demonstrated human pathologist performance in cancer detection and classification [17, 18]. Further than replicating the work of a pathologist, deep learning solutions might also be able to predict key molecular markers directly from H&E stained WSIs [19–21]. Recently, there has been some initial attempts to predict gene expression assays directly from breast cancer WSIs, both of larger gene sets for proliferation scores [22], as well as of the aforementioned 11-gene proliferation score [23]. We have previously shown that the mRNA expression of 17,695 genes could be predicted from WSIs, and used the 11-gene score predicted individually as a proof of concept that the genes’ expression were correctly predicted [20]. However, whether these predicted gene scores are associated with prognosis and patient survival remains to be determined. In this study we use an in-house dataset together with public data from The Cancer Genome Atlas (TCGA) to train two deep learning models for prediction of molecular proliferation signatures: one for the 11-gene proliferation score, and one for the individual MKI67 proliferation marker. We then validate the models on a third fully independent non-public external dataset to assess the predictions and their generalisability, which has not been done in the past. Furthermore, we evaluate the prognostic performances of the two proliferation indicators.

## Methods

### Datasets

Female patients with primary invasive breast cancer tumours who underwent surgery without neoadjuvant chemotherapy from three different cohorts were included: the in-house dataset Clinseq breast cancer cohort [24, 25] (*n* = 270), and the publicly available TCGA breast cancer cohort [6] (*n* = 721) were used for training, and the non-public SCAN-B Lund data (*n* = 997) was used as a fully external validation cohort [26, 27]. The Clinseq study includes both patients diagnosed in 2012 at Stockholm South General Hospital and patients who had surgery between 2001 and 2008 at Karolinska University Hospital in Stockholm, Sweden. For cases from the TCGA cohort, information was retrieved from the American Cancer Genome Atlas study, which is a publicly available database including both pathology and molecular data. Thirdly, the SCAN-B study consists of patients diagnosed between 2010 and 2019 at Lund University Hospital in Lund, Sweden, from which *n* = 997 patients were eligible in this study (see Additional Figs. 1 and 2 for consort diagrams). All three datasets contain H&E stained histopathological slides scanned at 40x magnification (0.23 μm pixel size) into digital WSIs and normalised RNA-seq data of gene expression levels of all 20,477 protein encoding genes as well as clinical data, stage, biomarker status and histologic NHG grade for each patient. Additional Table 1 presents the patient characteristics of cases included in the training set, internal and external test sets.

### WSI pre-processing

The WSIs were pre-processed according to procedures previously described in Wang et al. [27]. Briefly, tissue segmentation was first performed by using OpenSlide to extract lower-level representations from the WSIs resolution pyramids [28].These were then transformed from RGB to HSV colour space and two masks were generated to remove non-tissue areas while reducing the removal of tissue regions. Next, WSIs were divided into image tiles of 598 × 598 pixels with a resolution of 20 × (271 × 271 μm). During tiling, the stride was set to 50% overlap between two consecutive tiles. To accommodate for stain and scanner variability, the WSIs were colour normalised according to the method described in Macenko et al. [29], and adapted in Wang et al. with a slide level colour normalisation [27]. Next, only tumour regions were selected by using a CNN model previously developed [27]. Finally, to ensure quality of the data, blurred tiles were detected by measuring the variance after Laplacian filtering from the OpenCV package with kernel size set to 1 and tiles with a value lower than 500 were excluded [30].

### RNA-seq data preparation

The preparation of the RNA-seq values was performed according to the methods described previously [20, 25, 26]. The Clinseq and TCGA cohorts were merged for training. RNA-seq values from Clinseq were normalised on a gene level to have a median value equal to the data from TCGA [20]. SCAN-B data were preprocessed using several library protocols and were normalised across all protocols according to the methodology previously described [26].

### Molecular definition

Two markers of proliferation were used in this study: one 11-gene set extracted from the PAM50 gene expression assay [4], and one proliferation marker: MKI67. The 11-gene set (hereafter referred to as the 11-gene proliferation score) is defined as the mean of the normalised expressions of the 11 genes BIRC5, CCNB1, CDC20, CEP55, MKI67, NDC80, NUF2, PTTG1, RRM2, TYMS and UBE2C which are proliferation-associated genes [4, 12, 23]. The 11-gene proliferation score is obtained here as the average of the scores for each of the 11 genes. MKI67 itself is individually highly correlated with many proliferation signatures [15]. The proliferation score and MKI67 were assigned as the respective outcomes for the models to predict for all tiles of each patient.

### Model training

In order to train our CNN models, the *n* = 991 study patients (Clinseq + TCGA) and their corresponding WSIs were split into different datasets. A majority of the patients (83%, *n* = 819) were arranged into training (60%), tuning (20%) and validation (20%) datasets, with an even distribution of breast cancer subtypes between the datasets, for model optimisation and pre-evaluation. An internal test set of 172 randomly selected patients (about 17% from each dataset) was kept untouched throughout the model optimisation.

The 48 layer deep CNN architecture Inception v3 [31] was implemented in Python using Keras with TensorFlow backend and GPU hardware (Nvidia RTX 2080 ti). The image tiles (and particularly each pixel in the tiles) were used as features for the prediction. The response variables used were the patient-level gene expression of the mean of the 11 genes’ expressions for the proliferation score, or the expression of the MKI67 gene. The output layer of the CNN model was replaced by one neuron and a linear activation to build a regression model and address a univariate continuous response as both the proliferation score and the MKI67 score are a continuous value. The weights were initialised from an ImageNet pre-trained model. The model was optimised by means of backpropagation using mini-batch gradient descent with mean squared error (MSE) as the loss function and Keras’ Adam optimiser with default values except for a learning rate value of 1 × 10^− 6^.

The model was optimised separately for predicting the 11-gene proliferation score and the MKI67 marker iteratively over partial epochs, in each epoch 400 mini-batches of n = 12 tiles each stochastically selected from the training dataset. The loss score MSE was calculated for each mini-batch as the mean squared difference between predictions (Y’) and true targets defined by RNA-seq (Y). In addition, the training MSE was found for all 400 mini-batches of each partial epoch. Data augmentation of tiles (rotating, mirroring and flipping) was applied during model optimisation. An early stopping criterion with patience of 50 epochs was used or until 500 epochs were reached. The best models were then assessed on the validation dataset (163 patients) and the prediction performance on this unseen data was used as a criterion when selecting the best performing learning rate. The corresponding two models were retrained using all the training data and then evaluated on the internal test set (172 patients). Lastly, the final models were also evaluated on the fully independent external test set of *n* = 997 patients.

### Model accuracy

The median value of the predictions among all tiles of a given WSI was selected as the patient level predicted proliferation score or marker, and plotted against the RNA-seq based values which the models were trained to predict. This allowed correlation analysis using linear regression (R^2^) and the rank-based non-parametric Spearman rank correlation coefficient (*ρ*) to be estimated with a corresponding p-value where *p* < 0.05 was considered significant. Additional analyses were performed for different patient subgroups: by Estrogen receptor status (ER); defined as positive when at least 10% of cells stain positive in the patient’s resected tumour, by NHG status, and by cancer subtype.

### Relation between predictions and ER status, NHG and breast cancer subtypes

The relationships between RNA-seq based and CNN predicted proliferation score or MKI67 and ER status, NHG and breast cancer subtype were assessed in the internal test set and the external test set using boxplots. Independent t-tests were performed to assess group-wise comparisons in predicted values between risk groups.

### Prognostic value of CNN model-based predictions

Recurrence-free survival (RFS), as defined as the time until the occurrence of a locoregional or distant metastasis, contralateral tumour, or death, was compared between patients’ risk groups. For both the predicted 11-gene proliferation score and the proliferation marker, patients were dichotomized into a low- and high-risk group using the median value in the total patient population in the external test set. Kaplan-Meier curves were used to visualise different outcomes between groups, and multivariate Cox proportional hazard modelling was used to estimate hazard ratios (HR) adjusting for patient age, tumour size (< 20 mm or ≥20 mm), lymph node status, status of routine biomarkers (ER, and Human Epidermal Growth Factor Receptor 2 (HER2)), and NHG, and establishing a 95% confidence interval (CI). Analyses were performed for the entire external test set, as well as by ER subgroup.

## Results

**Fig. 1:**
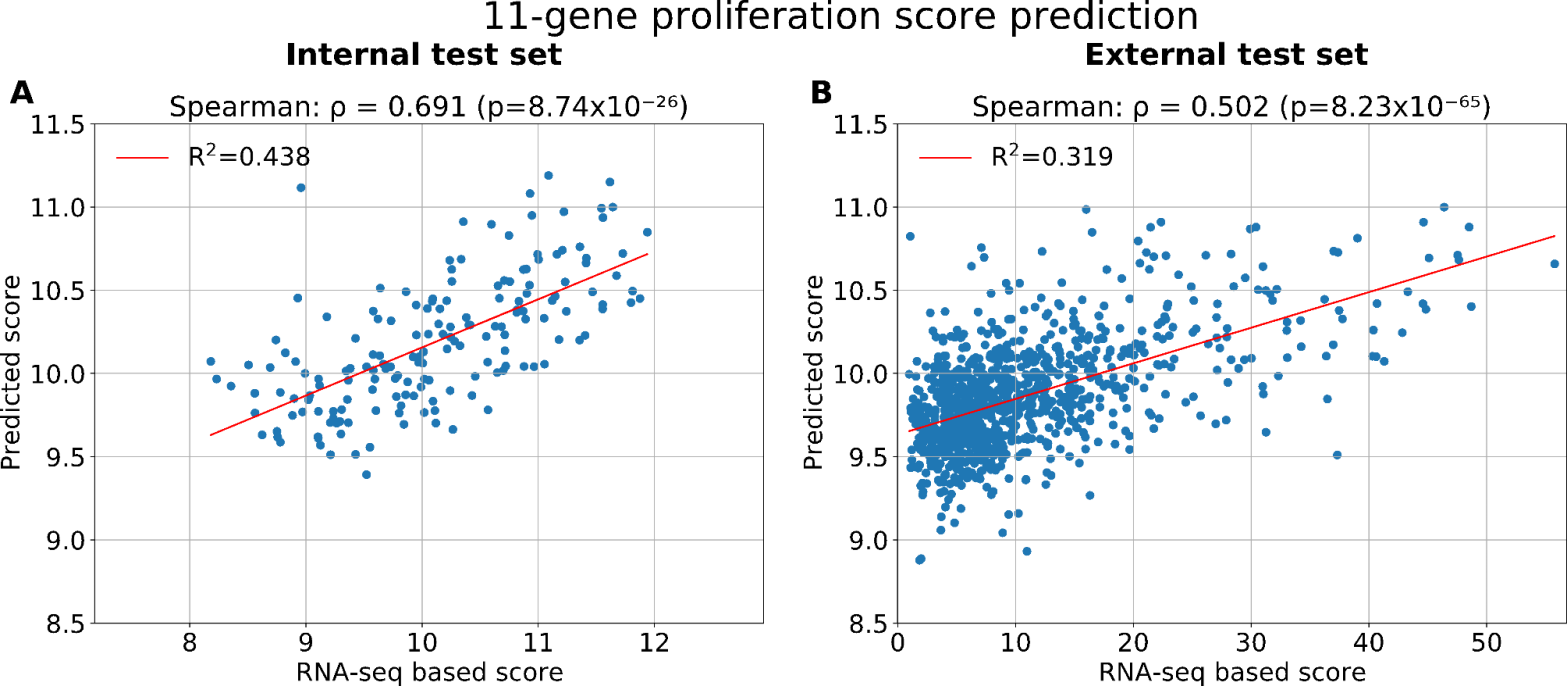
Model performance for the 11-gene proliferation score prediction Scatterplots of the 11-gene proliferation score predictions and RNA-seq values. **(A)** Results for the internal test set of TCGA and ClinSeq patients (*n* = 172). **(B)** Performance on the external test set from SCAN-B (*n* = 997).

**Fig. 2:**
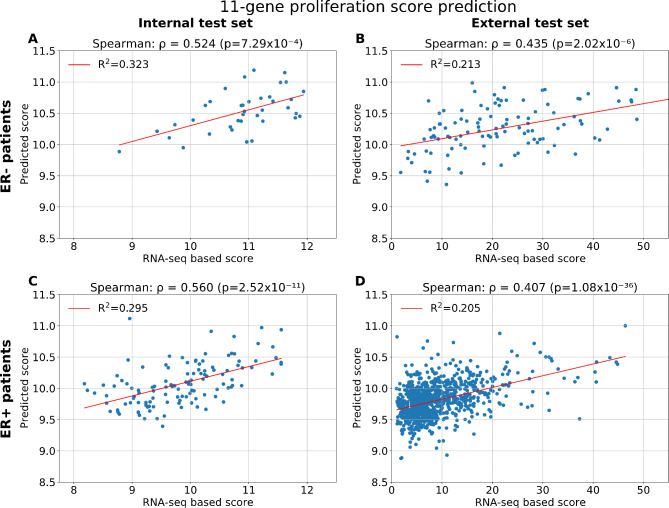
Model performance for the 11-gene proliferation score prediction by patients’ ER status Scatterplots of the 11-gene proliferation score predictions and RNA-seq values. **(A)** Results for the internal test set, ER- patients (*n* = 38) **(B)** Results for the internal test set, ER + patients (*n* = 121) **(C)** Results for the external test set, ER- patients (*n* = 110) **(D)** Results for the external test set, ER + patients (*n* = 885).

To predict the 11-gene proliferation score expression levels directly from WSIs, a deep CNN model was optimised. In the cross-validation results (819 patients from TCGA + ClinSeq datasets), a Spearman correlation coefficient of *ρ* = 0.704 (*p*-value < 0.001) and an R^2^ value of 0.414 were obtained for the correlation between the predicted proliferation score and the corresponding RNA-seq derived score (data not shown). In the independent unseen internal test set (TCGA + ClinSeq) of 172 patients, the model for the 11-gene proliferation score predictions has a performance of *p* = 0.691 (with *p* < 0.001) and R^2^ = 0.438 (Fig. 1A). The model was then evaluated on 997 patients from the unseen external test set (SCAN-B). The obtained performance of *ρ* = 0.502 (*p* < 0.001) and R^2^ = 0.319 is presented in Fig. 1B. Further analyses were performed on different patient subgroups. Figure 2(A-B) presents results for the ER-negative (ER-) patient subgroup while Fig. 2(C-D) presents results for the ER-positive (ER+) patients. In the external test set, the obtained performance was *ρ* = 0.435 (*p* < 0.001) and *p* = 0.407 (*p* < 0.001) for ER- and ER + patients subgroups respectively. A significant spearman correlation was obtained across patient subtypes (Additional Fig. 3(E-H)) as well as across patient grades (Additional Fig. 4(D-F)).

**Fig. 3:**
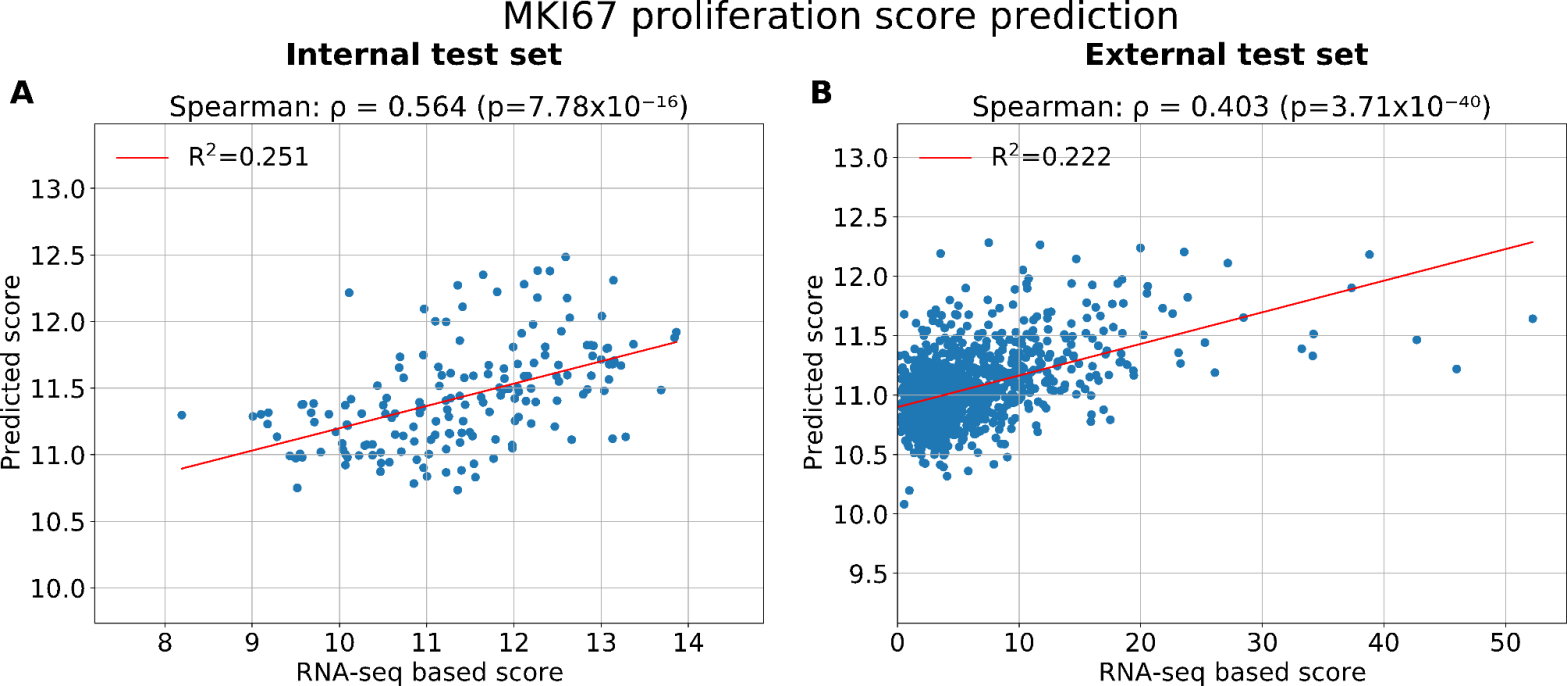
Model performance for the MKI67 proliferation marker prediction Scatterplots of the MKI67 proliferation gene predictions and RNA-seq values. **(A)** Results for the internal test set TCGA + ClinSeq patients (*n* = 172). **(B)** Performance on the external test set of SCAN-B patients (*n* = 997).

**Fig. 4:**
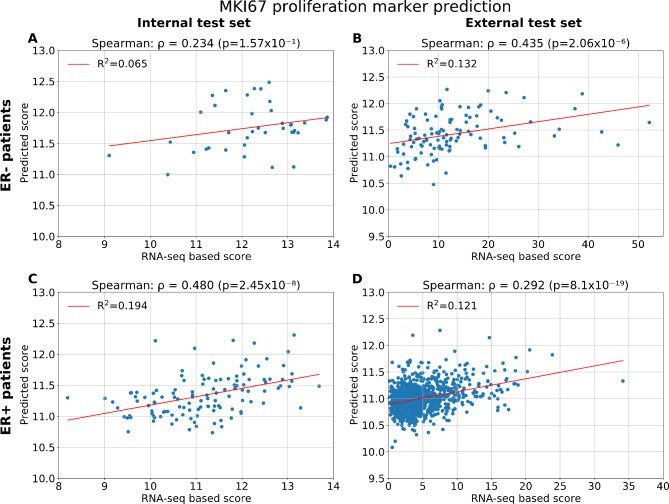
Model performance for the MKI67 proliferation marker prediction by patients’ ER status Scatterplots of the MKI67 proliferation marker predictions and RNA-seq values. **(A)** Results for the internal test set, ER- patients (*n* = 38) **(B)** Results for the internal test set, ER + patients (*n* = 121) **(C)** Results for the external test set, ER- patients (*n* = 110) **(D)** Results for the internal test set, ER + patients (*n* = 885)

The MKI67 single gene expression was also predicted and evaluated as it is an established marker for proliferation. The performance obtained in the cross-validation subset was a Spearman correlation coefficient of *ρ* = 0.654 (*p* < 0.001) and R^2^ = 0.324 (data not shown). Performance on the unseen internal test set was *ρ* = 0.564 (*p* < 0.001) and R^2^ = 0.251 (Fig. 3A), while in the external test set the performance obtained for the prediction of MKI67 gene expression was of *ρ* = 0.403 (with *p* < 0.001) and R^2^ = 0.222 (Fig. 3B). For ER- patients in the external test set, the performance obtained was *ρ* = 0.435 (with *p* < 0.001) (Fig. 4B), and *ρ* = 0.292 (with *p* < 0.001) for the ER + patients (Fig. 4D). For patient subtypes, the performance was significant within the Luminal B and Basal-like patients (Supplementary Fig. 5(E-H)), as well as for grade 3 patients (Supplementary Fig. 6(D-F)).

**Fig. 5:**
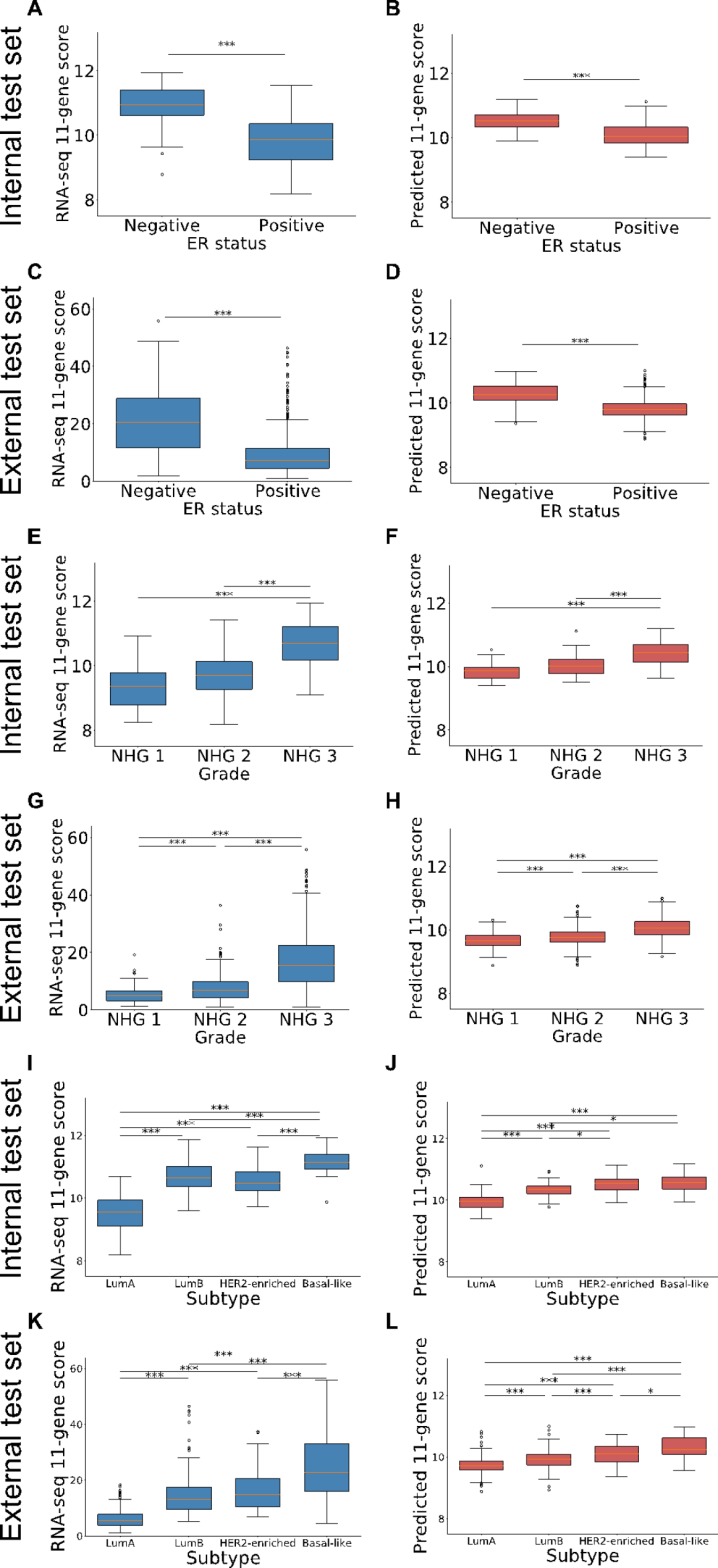
11-gene proliferation score by ER status, grade and subtype Box-plot presenting RNA-seq values (blue) and predicted values (red) for the 11-gene set proliferation score by ER status, grade and subtype. **(A)** RNA-seq score by ER status in the internal test set **(B)** Predicted score by ER status in the internal test set **(C)** RNA-seq score by ER status in the external test set **(D)** Predicted score by ER status in the external test set **(E)** RNA-seq score by grade in the internal test set **(F)** Predicted score by grade in the internal test set **(G)** RNA-seq score by grade in the external test set **(H)** Predicted score by grade in the external test set **(I)** RNA-seq score by subtype in the internal test set. **(J)** Predicted score by subtype in the internal test set **K)** RNA-seq score by subtype in the external test set **L)** Predicted score by subtype in the external test set. *p-value < 0.05, **p-value < 0.01, ***p-value < 0.001.

**Fig. 6:**
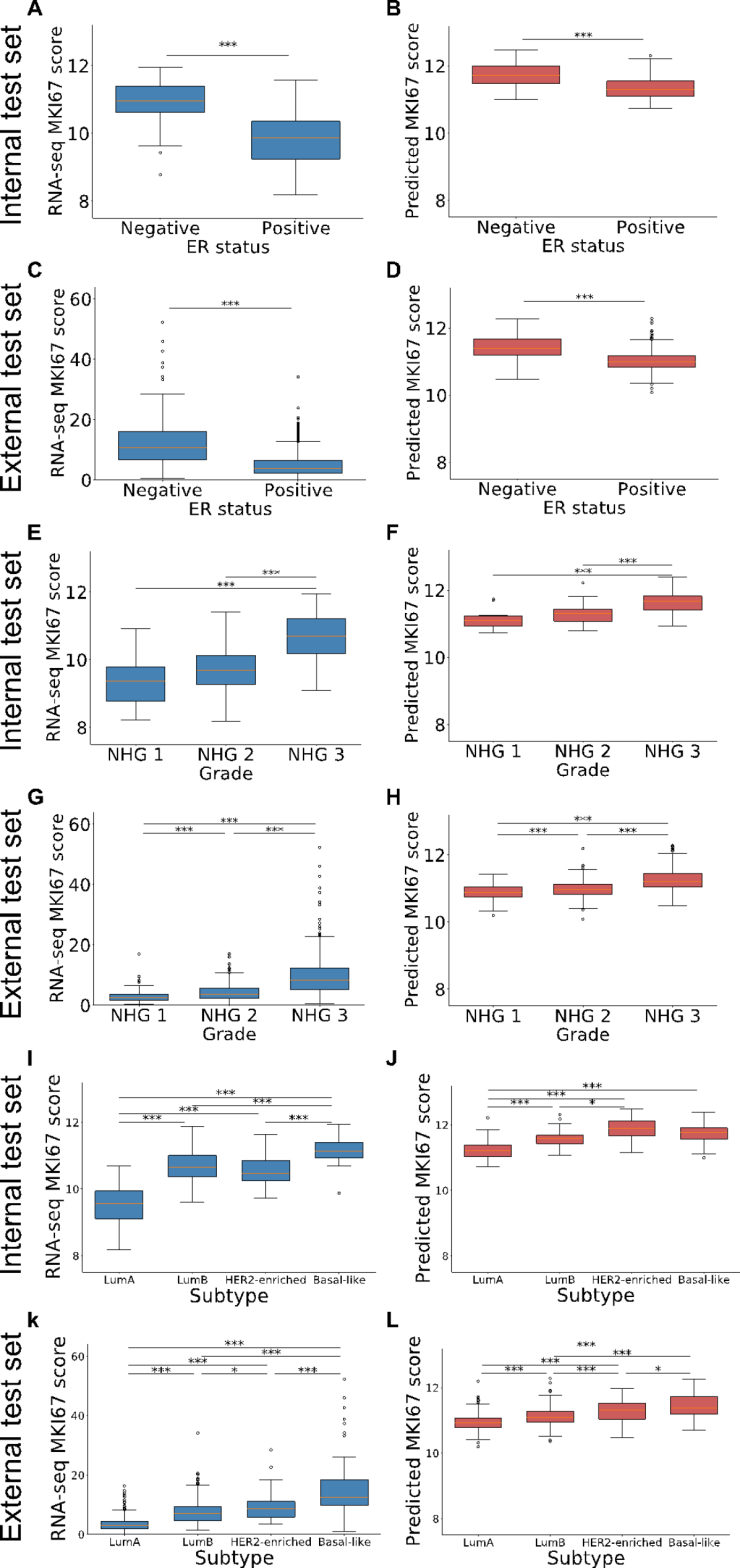
MKI67 proliferation marker by ER status, grade and subtype Box-plot presenting RNA-seq based marker values (blue) and predicted marker values (red) for the MKI67 proliferation marker by ER status, grade and subtype. **(A)** RNA-seq score by ER status in the internal test set **(B)** Predicted score by ER status in the internal test set **(C)** RNA-seq score by ER status in the external test set **(D)** Predicted score by ER status in the external test set **(E)** RNA-seq score by grade in the internal test set **(F)** Predicted score by grade in the internal test set **(G)** RNA-seq score by grade in the external test set **(H)** Predicted score by grade in the external test set **(I)** RNA-seq score by subtype in the internal test set. **(J)** Predicted score by subtype in the internal test set **K)** RNA-seq score by subtype in the external test set **L)** Predicted score by subtype in the external test set. *p-value < 0.05, **p-value < 0.01, ***p-value < 0.001

The relationships between gene expression levels and other prognostic factors, particularly breast cancer grade (NHG 1, 2 and 3) and subtype (Luminal A, Luminal B, Basal-like and HER2-enriched) as well as ER status were evaluated for both the predicted levels and the RNA-seq values. Figure 5 presents the results for the 11-gene proliferation score variations by grade and subtype in both the internal and external test sets. Figure 6 shows similar results for the MKI67 proliferation gene. Overall, RNA-seq based values (blue boxes) show larger variance among patients compared to predicted values (red boxes). Moreover, similar trends regarding the proliferation scores by ER status, grade and subtype were observed across RNA-seq based values and predicted values. ER-negative patients had higher proliferation score and marker values than ER-positive patients. NHG 3 patients had the highest proliferation score and marker values while NHG 1 patients had the lowest values. Regarding subtypes, Luminal A patients consistently had the lowest levels of proliferation, both in terms of the 11-gene set and with the MKI67 marker.

**Fig. 7:**
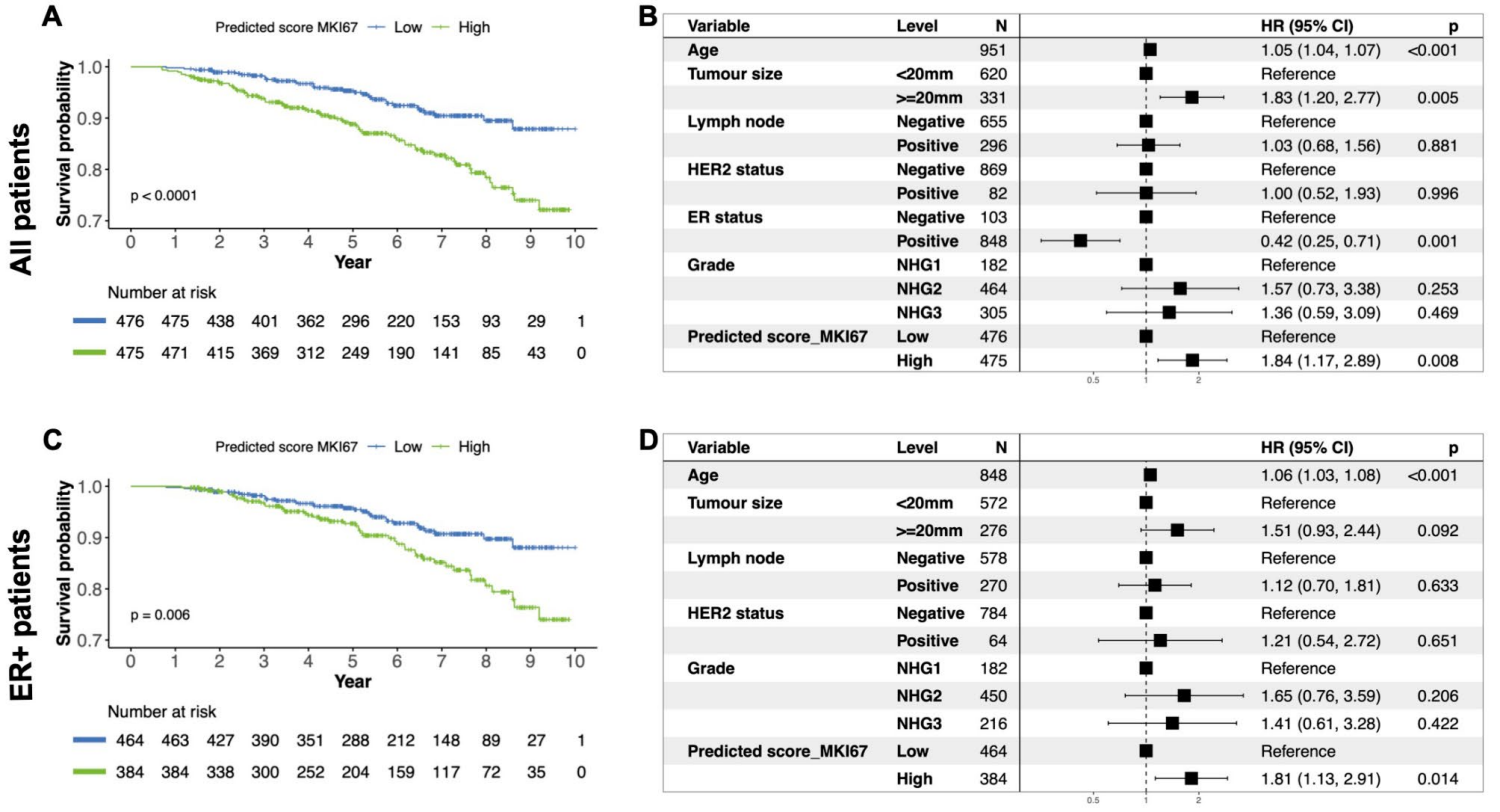
Prognostic performance of the predicted 11-gene set proliferation score Evaluation of the prognostic performance on recurrence-free survival (defined as the time to having a locoregional or distant metastasis, contralateral tumour or death) in the external SCAN-B dataset. **(A)** Kaplan-Meier curve for all patients stratified by the predicted 11-gene proliferation score into high- and low-risk groups. **(B)** Forest plot from multivariable Cox proportional hazard regression in all patients for the predicted 11-gene proliferation score based risk groups, adjusting for age, tumour size, lymph node status, grade, ER status and HER2 status. **(C)** Kaplan-Meier curve for ER-positive patients stratified by the predicted 11-gene proliferation score into high- and low-risk groups. **(D)** Forest plot from multivariable Cox proportional Hazard regression in ER-positive patients for the predicted 11-gene proliferation score based risk groups, adjusting for age, tumour size, lymph node status, grade and HER2 status

The prognostic performance of both the 11-gene proliferation score and the MKI67 proliferation marker were then evaluated in the external SCAN-B test set by time-to-event analyses. For the 11-gene score, we observe from the Kaplan-Meier curves that patients in the high-risk group had a worse prognosis, both when considering all patients (Fig. 7A) and the clinically relevant subgroup of ER-positive patients (Fig. 7C). Using univariate Cox proportional hazard regression, when including all patients, those in the high-risk group had an estimated hazard ratio of 2.08 (p-value < 0.001, 95% CI: 1.38;3.13) of recurrence compared to the low-risk group. The 11-gene proliferation score was also found to be a predictor of recurrence-free survival with an estimated hazard ratio of 1.65 (p-value = 0.03, 95% CI: 1.05; 2.61) for patients in the high-risk group, independently of patients’ age, tumour size, lymph node status, ER status, HER2 status or grade (Fig. 7B). Similar results were obtained in patients in the ER-positive subgroup with an obtained adjusted hazard ratio of 1.63 (p-value = 0.04, 95% CI: 1.01;2.63) (Fig. 7D).

**Fig. 8:**
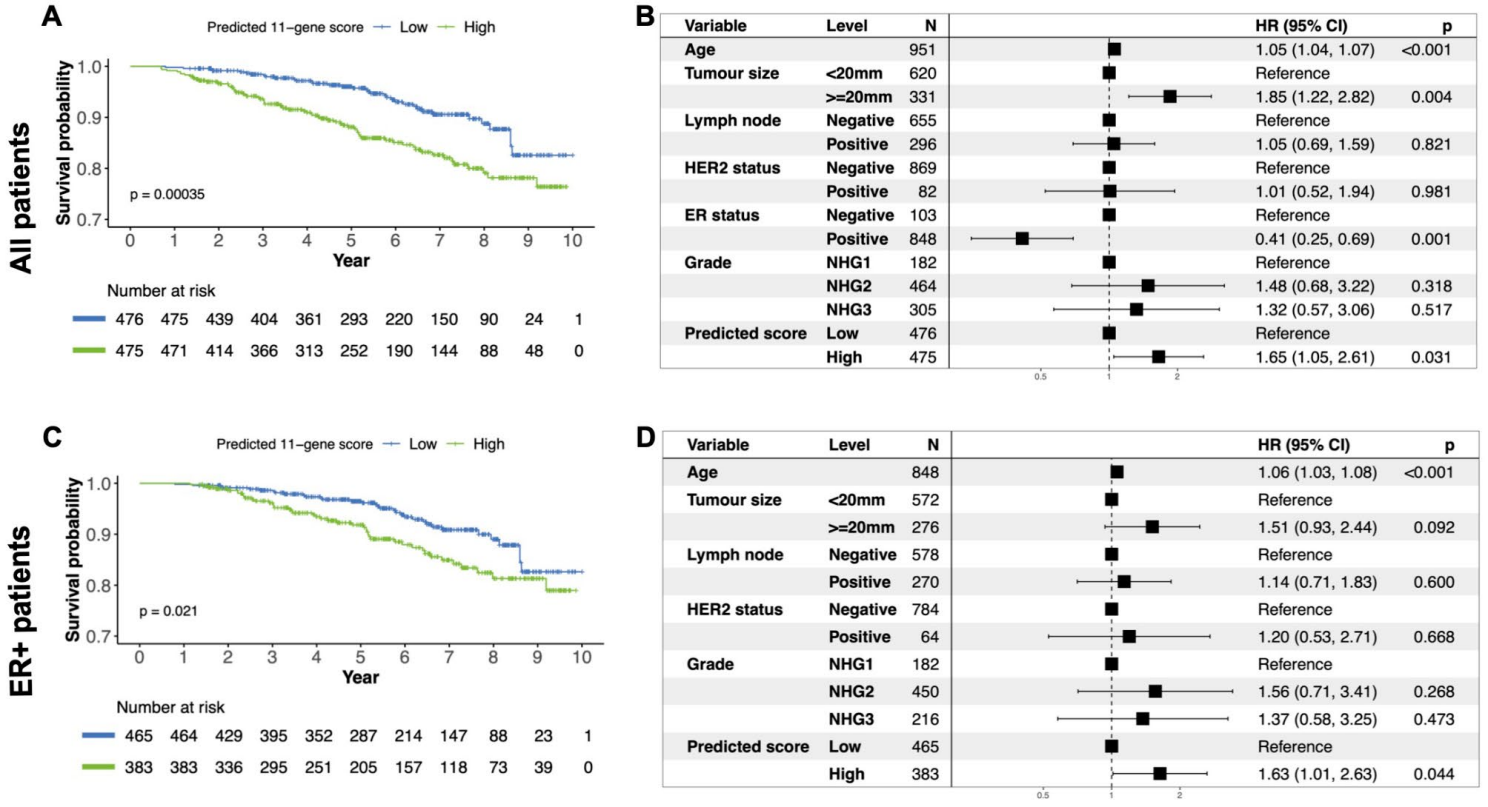
Prognostic performance of the predicted MKI67 gene proliferation marker Evaluation of the prognostic performance on recurrence-free survival (defined as the time to having a locoregional or distant metastasis, contralateral tumour or death) in the external SCAN-B dataset. **(A)** Kaplan-Meier curve for all patients stratified by the predicted MKI67 gene proliferation marker into high- and low-risk groups. **(B)** Forest plot from multivariable Cox proportional hazard regression in all patients for the predicted MKI67 gene proliferation marker based risk groups, adjusting for age, tumour size, lymph node status, grade, ER status and HER2 status. **(C)** Kaplan-Meier curve for ER-positive patients stratified by the predicted MKI67 gene proliferation marker into high- and low-risk groups. **(D)** Forest plot from multivariable Cox proportional Hazard regression in ER-positive patients for the predicted MKI67 gene proliferation marker risk groups, adjusting for age, tumour size, lymph node status, grade and HER2 status

Similar analyses were performed using the MKI67 gene proliferation marker. The Kaplan-Meier curves show that the gene expression prediction can identify patients with a higher risk of recurrence, both within all patients (Fig. 8A) and in the ER-positive subgroup (Fig. 8C). When including all patients, the multivariable Cox Proportional hazards model adjusting for age, tumour size, lymph node status, HER2 status, ER status and grade estimated a hazard ratio of 1.84 (p-value = 0.008, 95% CI: 1.17;2.89) for patients in the high-risk group compared to those in the low-risk group (Fig. 8B). Similar results were obtained in the ER-positive subgroup of patients with an adjusted hazard ratio of 1.81 (p-value = 0.01, 95% CI: 1.13; 2.91) (Fig. 8D).

## Discussion

In this study we show that the 11-gene proliferation score and the individual proliferation marker MKI67 can be successfully predicted using a CNN model and routine H&E stained breast cancer histopathology slides. All the results were validated on an external, fully independent and non-public dataset. The correlation between the predicted levels and the RNA-seq expression levels, were significant (*p* < 0.05) in both the internal and external test sets. This was also true in both ER-negative and ER-positive groups of patients. Moreover, the score and marker (RNA-seq and predicted) showed expected association with tumour ER status, grade (NHG) and molecular subtype for both the internal and external datasets. More importantly, we found that both the proliferation score and the proliferation marker were significantly associated with prognostic performance in the external set, even after adjusting for other clinical variables. This held true in the whole cohort as well as in the ER-positive subgroup in which treatment decisions can be difficult to determine using routine slides only.

Proliferation is known to be associated with prognosis [32, 33]. Most efforts for automatic assessment of proliferation have focused on mitosis detection [34], while a challenge from 2019 focused on the prediction of the 11-gene proliferation score [23]. This challenge only included one dataset (TCGA) split between a training and test set. The best team obtained a Spearman correlation of 0.617, lower than the obtained correlation for our internal test set of 0.691. It is of note that to obtain that correlation, the winning team of the challenge relied on mitosis counting prior to the prediction of the proliferation score [23]. This could be an indication that additional features could be added to the model to potentially improve the obtained results. The results obtained in our internal test set are also better than those obtained previously on the same data using the mean of individually predicted scores for each of the 11 genes [20]. In addition, the new score suggested in this study would be more computationally efficient as it would require the training of only one model as opposed to 11 separate models. With regards to the MKI67 marker, Mondol et al., showed that gene expression prediction is not as strong as for other genes, obtaining a correlation lower than 0.490 in their internal test set, compared to 0.564 in this study [22]. The main limitation of using Ki-67 protein assessment using IHC-based analyses is the high inter- and intra-observer variation which hinders its clinical utility [35]. The measurement of the proliferation marker MKI67 could overcome this limitation [20]. As previously shown, higher values in both the MKI67 proliferation marker and the 11-gene proliferation score were associated with a higher grade [2, 22], as well as with a negative ER status [36].

In terms of clinical value, not only have we shown that the proliferation score (the 11-gene set) and marker (MKI67) could be predicted using WSIs, we have also demonstrated that they have high prognostic value which can guide clinical management and predict patient outcome. RNA-seq levels of the PAM50 genes of which both the 11-gene set and the MKI67 proliferation marker are part, have previously been shown to be associated with prognosis [12], but it is the first time that their respective predicted levels using only WSIs are demonstrated to also be associated with prognosis. Several studies based on RNA-seq have shown that adding gene signature markers to the existing PAM50 intrinsic subtypes could assist in improving risk prediction [12, 37], it is possible that this is also the case with WSI predictions. In particular, we identified a subgroup of patients with higher proliferation scores in the ER-positive subgroup which had higher recurrence rates which could have clinical implications. In addition, compared to gene expression assays such as the PAM50 approach, the suggested solution is cost-effective and would prevent long waiting times for both the pathologists and the patients as it takes advantage of the already existing WSIs. We have also proven our methodology on a completely independent non-public external dataset in addition to the unseen internal test set, strengthening the obtained results. Furthermore, the advantage of using RNA-seq as the ground truth is that this method is independent from pathologists’ assessments.

There are some limitations that should be mentioned. Firstly, a weakly-supervised approach was used with only one label on a patient level for the proliferation score assigned to all of the tiles from that patient. Genes are likely expressed differently in the tumour presenting tumour heterogeneity with different scores obtained for different areas of the tumour [19]. In the future, it could be interesting to identify specific tumour regions where proliferation scores are over-expressed which could have an insight clinically but also for research purposes in order to find targeted treatments. The current training set included 819 patients, in the future increasing the number of patients could be beneficial to obtain higher performance. Finally, the RNA-seq values used in the training and testing set could not be matched. It is possible that the correlation of the predicted results with the external RNA-seq values could be even higher in the future.

## Conclusions

The results from this study suggest that the gene expression levels of both the 11-gene proliferation score and the MKI67 proliferation marker can be predicted directly from breast cancer morphology in routine histopathology slides using deep learning in a computationally efficient manner and in a fully independent test set. Both predicted scores were also found to be significantly associated with a higher risk of recurrence. This could have implications for identifying high risk patients, with applications in both cancer research and in clinical settings, where novel decision support to help in identifying patients that could benefit from more aggressive treatments is needed. AI-based solutions to assess risk of recurrence have the benefit of lower cost and short lead times compared with molecular assays.

## Electronic supplementary material

Below is the link to the electronic supplementary material.

Supplementary Material 1

## Data Availability

The datasets analysed during the current study are not publicly available due to local privacy laws but are available from the corresponding author upon reasonable request.

## Abbreviations

WSI: Whole Slide Images
CNN: Convolutional Neural Network
HR: Hazard ratio
H&E: Haematoxylin and Eosin
NHG: Nottingham Histologic Grade
IHC: Immunohistochemistry
TCGA: The Cancer Genome Atlas
MSE: mean squared error
ER: Estrogen receptor
HER2: Human Epidermal Growth Factor Receptor 2
CI: Confidence interval
RFS: Recurrence-free survival
