# Supplementary material for: Prediction of gene expression-based breast cancer proliferation scores from histopathology whole slide images using deep learning
Source: BMC Cancer. 2024 Dec 11;24:1510. doi: 10.1186/s12885-024-13248-9 (PMC11633006; doi:10.1186/s12885-024-13248-9)
Supplement: Supplementary file 1 — Supplementary Material 1 [file 12885_2024_13248_MOESM1_ESM.pdf]

Supplementary Material

Prediction of gene expression-based breast cancer proliferation scores from histopathology whole slide images using deep learning

Andreas Ekholm[1], Yinxi Wang[1], Johan Vallon-Christersson[2], Constance Boissin[1#], Mattias Rantalainen[1,3#*]

[1]Department of Medical Epidemiology and Biostatistics, Karolinska Institutet, Stockholm, Sweden

[2] Division of Oncology, Department of Clinical Sciences Lund, Lund University, Lund, Sweden

[3]MedTechLabs, BioClinicum, Karolinska University Hospital, Stockholm, Sweden

[#]Equal contribution last authors.
*Corresponding author: mattias.rantalainen@ki.se

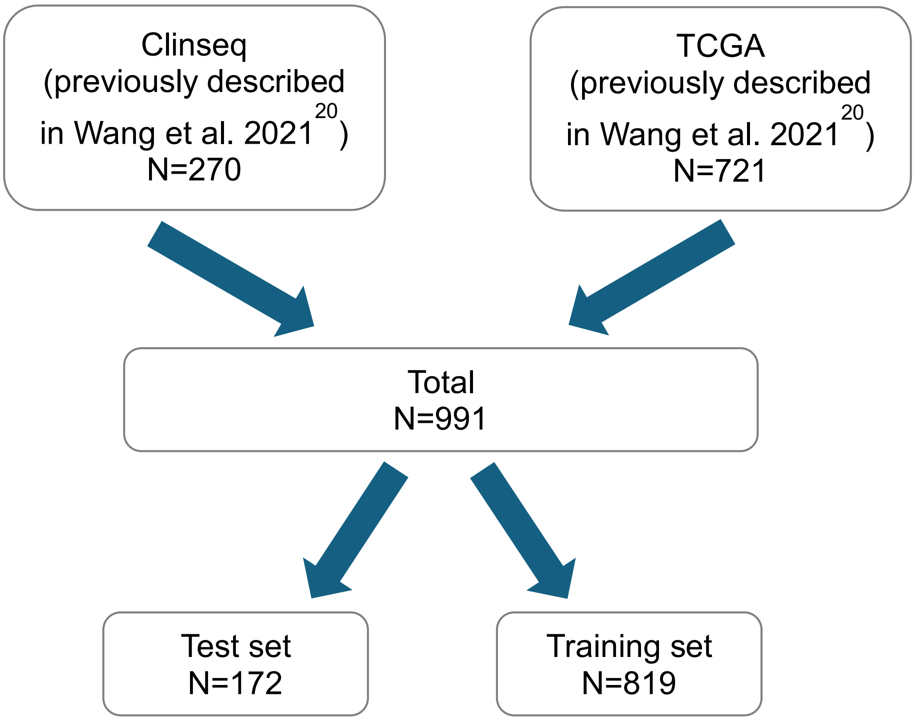

Additional Figure 1. Consort diagram of the training and internal test set. The training set included data from two cohorts the Clinseq cohort and the TCGA breast cancer cohort. Approximately 80% of each cohorts were kept aside as an independent test set.

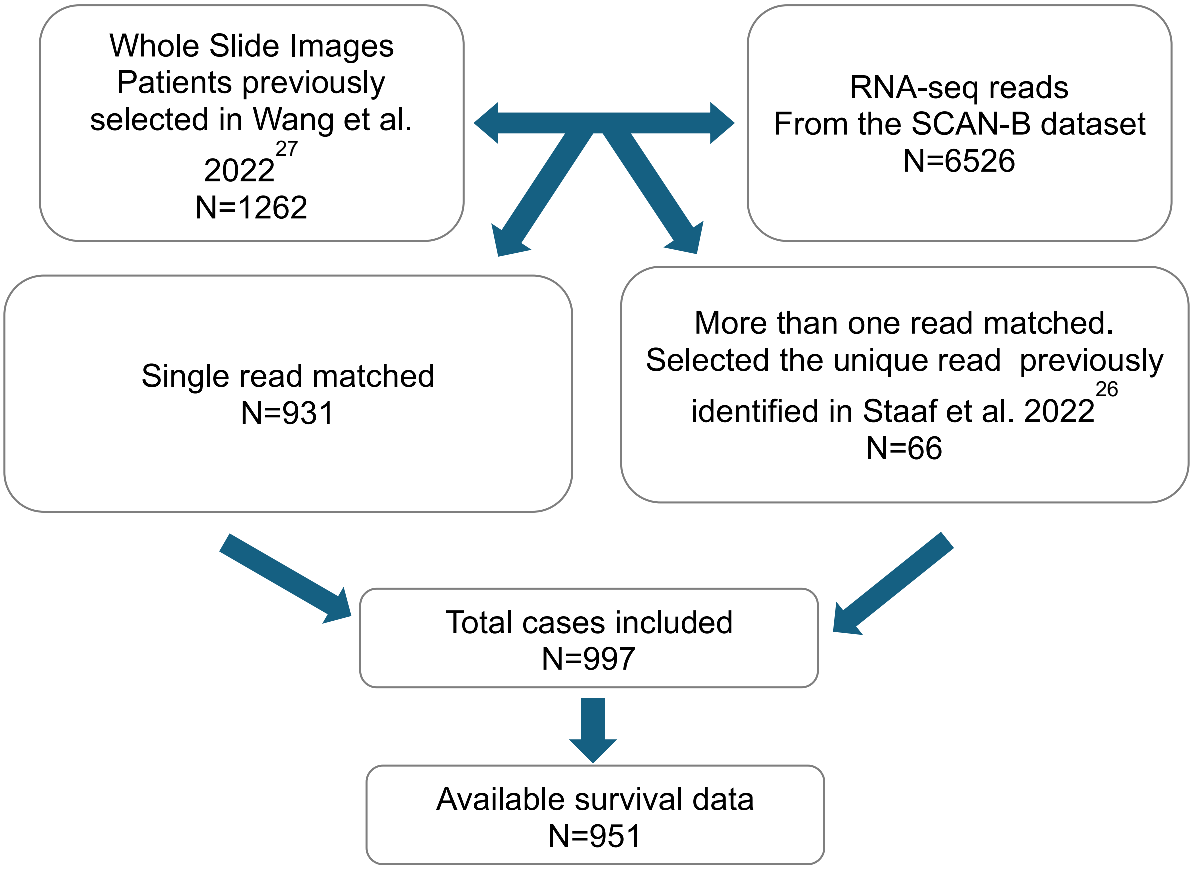

Additional Figure 2. Consort diagram of the fully independent test set. The external test set includes patients from the SCAN-B cohort for which whole slide images and RNA-seq reads had to be matched.

Additional Table 1. Clinical characteristics of patients included in the training set, internal test set and external test set

Dataset	Training set (Clinseq-TCGA)	Internal Test set (Clinseq-TCGA)	External Test set (SCAN-B)
N	819	172	997
Age (mean ±sd)	58.3 ± 12.7	57.4 ± 11.9	65.0 ± 12.5
Unknown (%)	121 (14.8)	55 (32.0)	0
Tumour size			
<20 mm (%)	272 (33.2)	39 (22.7)	649 (65.1)
>=20 mm (%)	522 (63.7)	100 (58.1)	338 (33.9)
Unknown (%)	25 (3.1)	33 (19.2)	10 (1.0)
Lymph node			
Negative (%)	475 (58.0)	70 (40.7)	667 (66.9)
Positive (%)	219 (38.9)	69 (40.1)	305 (30.6)
Unknown (%)	25 (3.1)	33 (19.2)	25 (2.5)
HER2 status			
Negative (%)	570 (69.6)	112 (65.1)	898 (90.1)
Positive (%)	100 (12.2)	21 (12.2)	86 (8.63)
Unknown (%)	149 (18.2)	39 (22.7)	13 (1.3)
ER status[a]			
Negative (%)	164 (20.0)	38 (22.1)	110 (11.0)
Positive (%)	614 (75.0)	121 (70.3)	885 (88.8)
Unknown (%)	41 (5.0)	13 (7.6)	2 (0.2)
Histological grade			
NHG 1 (%)	93 (11.4)	13 (7.6)	191 (19.2)
NHG 2 (%)	301 (36.8)	63 (36.6)	482 (48.3)
NHG 3 (%)	277 (33.8)	56 (32.6)	324 (32.5)
Unknown (%)	148 (18.1)	40 (23.3)	0
Cancer subtype			
Luminal A (LumA) (%)	384 (46.9)	74 (43.0)	514 (51.6)
Luminal B (LumB) (%)	150 (18.3)	30 (17.4)	203 (20.4)
Her2-enriched (%)	68 (8.3)	14 (8.1)	61 (6.1)
Basal-like (%)	105 (12.8)	28 (16.3)	73 (7.3)
Unknown (%)	112 (13.7)	26 (15.1)	146 (14.6)

sd: standard deviation
ER: estrogen receptor
NHG: Nottingham Histological Grade
[a]Positive if >=10% positively stained cells

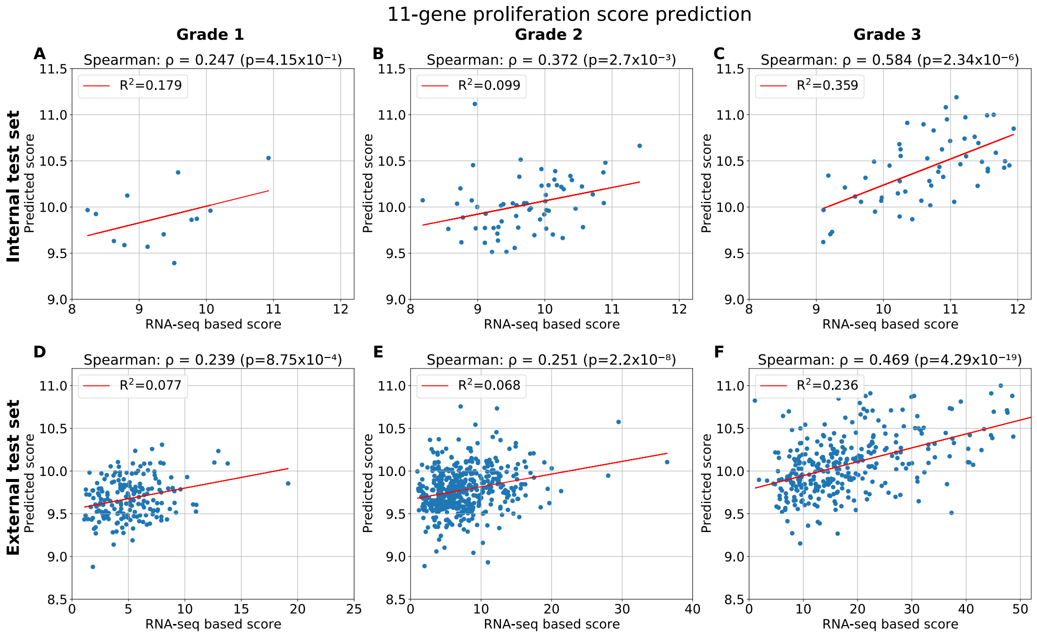

Additional Figure 3. Model performance for the 11-gene proliferation score prediction by patient NHG subgroup.

Scatterplots of the 11-gene proliferation score predictions and RNA-seq values. **A)** Results for the internal test set, NHG1 patients (n=13) **B)** Results for the internal test set, NHG2 patients (n=63) **C)** Results for the internal test set, NHG3 patients (n=56) **D)** Results for the external test set, NHG1 patients (n=191) **E)** Results for the external test set, NHG2 patients (n=482) **F)** Results for the external test set, NHG3 patients (n=324).

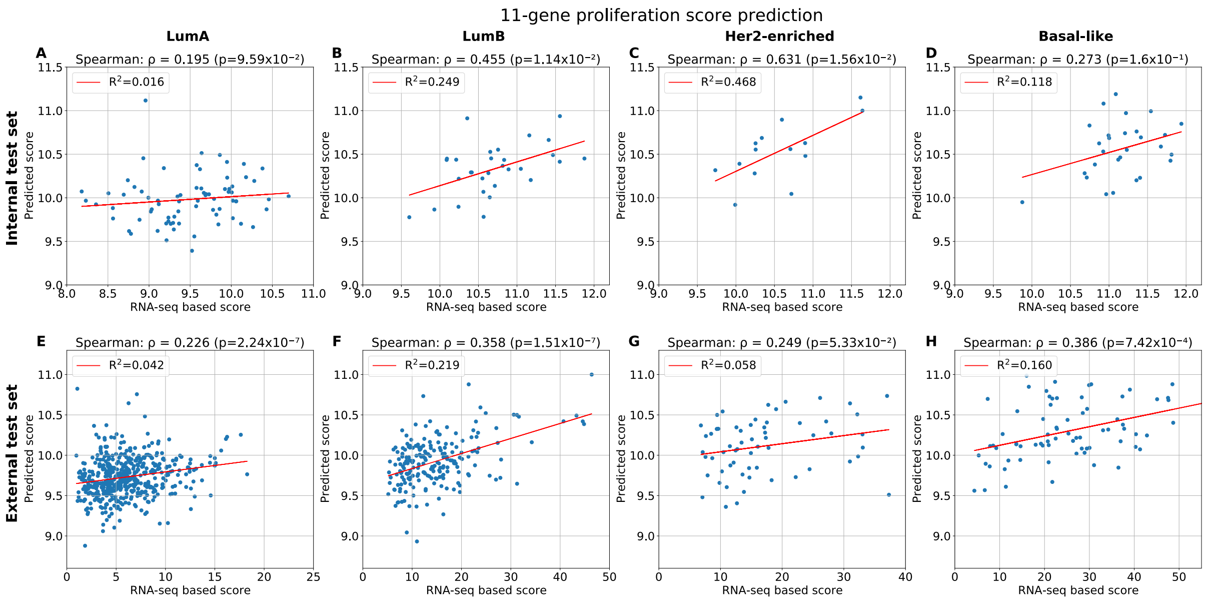

Additional Figure 4. Model performance for the 11-gene proliferation score prediction by patient subtype.

Scatterplots of the 11-gene proliferation score predictions and RNA-seq values. **A)** Results for the internal test set, patients with Luminal A subtype (n=74) **B)** Results for the internal test set, patients with Luminal B subtype (n=30) **C)** Results for the internal test set, patients with Her2-enriched subtype (n=14) **D)** Results for the internal test set, patients with Basal-like subtype (n=28) **E)** Results for the external test set, patients with Luminal A subtype (n=514) **F)** Results for the external test set, patients with Luminal B subtype (n=203) **G)** Results for the external test set, patients with Her2-enriched subtype (n=61) **F)** Results for the external test set, patients with Basal-like subtype (n=73).

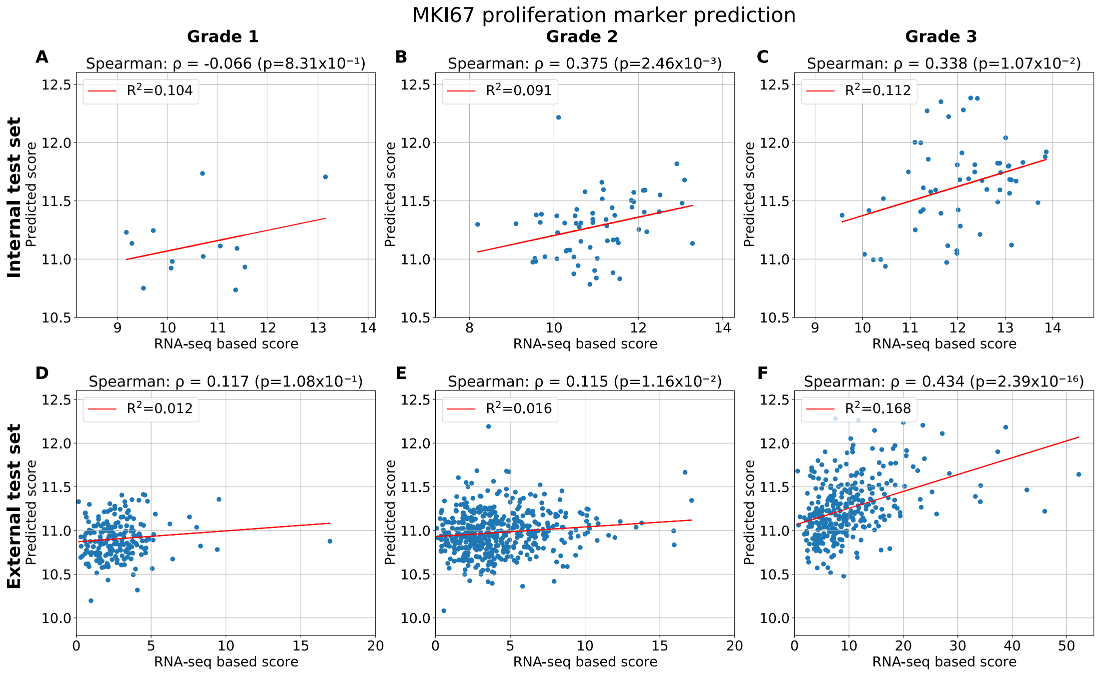

Additional Figure 5. Model performance for the MKI67 proliferation marker prediction by patient NHG subgroup.

Scatterplots of the MKI67 proliferation marker predictions and RNA-seq values. **A)** Results for the internal test set, NHG1 patients (n=13) **B)** Results for the internal test set, NHG2 patients (n=63) **C)** Results for the internal test set, NHG3 patients (n=56) **D)** Results for the external test set, NHG1 patients (n=191) **E)** Results for the external test set, NHG2 patients (n=482) **F)** Results for the external test set, NHG3 patients (n=324).

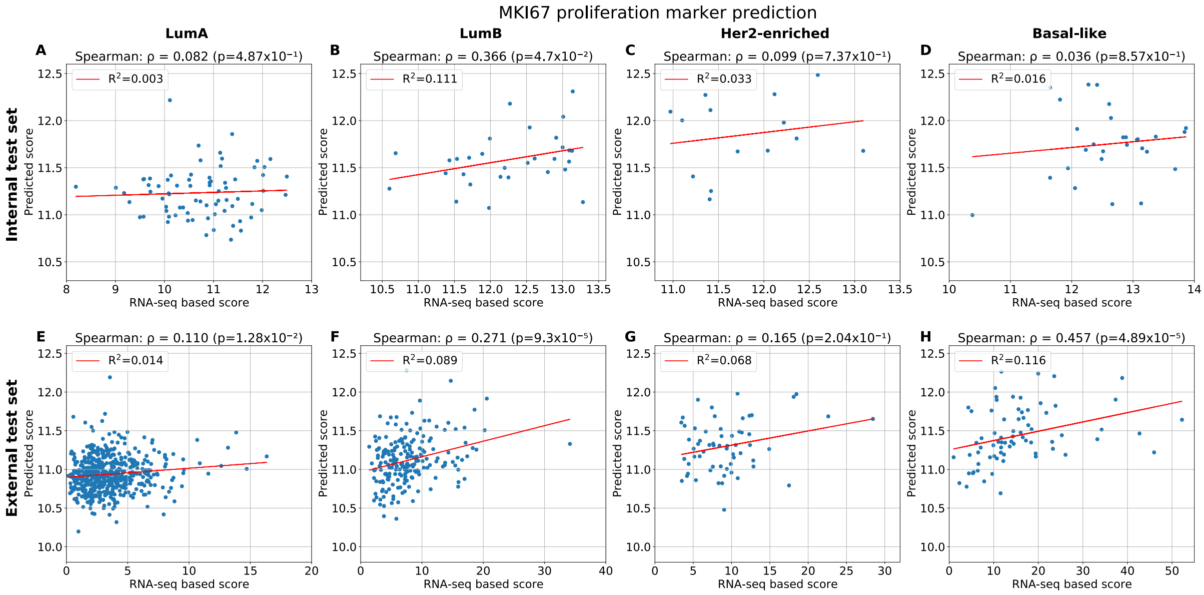

Additional Figure 6. Model performance for the MKI67 proliferation marker prediction by patient subtype.

Scatterplots of the MKI67 proliferation marker predictions and RNA-seq values. **A)** Results for the internal test set, patients with Luminal A subtype (n=74) **B)** Results for the internal test set, patients with Luminal B subtype (n=30) **C)** Results for the internal test set, patients with Her2-enriched subtype (n=14) **D)** Results for the internal test set, patients with Basal-like subtype (n=28) **E)** Results for the external test set, patients with Luminal A subtype (n=514) **F)** Results for the external test set, patients

with Luminal B subtype (n=203) **G)** Results for the external test set, patients with Her2-enriched subtype (n=61) **F)** Results for the external test set, patients with Basal-like subtype (n=73).
